# Unveiling Vacancy-Driven Stability: Atomic and Electronic Insights into Ni/Al_2_O_3_ Interfaces

**DOI:** 10.3390/molecules30214285

**Published:** 2025-11-04

**Authors:** Lili Duan, Renwei Li, Haifeng Yang, Dehao Kong

**Affiliations:** 1 College of Urban Rail Transit, Jilin Railway Technical University, Jilin 132299, China; 2 College of Mechanical and Electrical Engineering, Jilin Institute of Chemical Technology, Jilin 132022, China; 3 State Key Laboratory of Advanced Welding and Joining, Harbin Institute of Technology, Harbin 150001, China; 4 College of New Energy and Materials, Northeast Petroleum University, Daqing 163711, China

**Keywords:** metal/ceramic interface, vacancy defects, electronic structure

## Abstract

The Ni/Al_2_O_3_ interface bears the load transfer and energy dissipation, which determines the service performance of the composite materials. In this study, three distinct vacancy-defect-modified interface models (D_1_, D_2_, and D_3_, corresponding to vacancies in the first, second, and third layers of the Ni substrate surface, respectively) were constructed to systematically investigate the regulatory mechanism of vacancies on interfacial stability. The underlying mechanism of vacancy-enhanced interfacial stability was elucidated from both atomic-scale structural and electronic property perspectives. The results demonstrate that the D_1_, D_2_, and D_3_ structures increase the adhesion work of the interface by 2.0%, 6.7%, and 0.3%, respectively. This enhancement effect mainly stems from vacancy-induced atomic relaxation at the interface, which optimizes the equilibrium interfacial spacing and effectively releases residual strain energy. Further electronic structure analysis reveals a notable increase in charge density at the vacancy-modified interface (particularly in the D_2_ structure), indicating that vacancy defects promote charge transfer and redistribution by altering local electron distribution. More importantly, the bonding strength of the interface exhibits a positive correlation with electron orbital hybridization intensity, where stronger s-, p-, and d-orbit hybridization directly leads to a more stable interface. These findings provide atomic- and electronic-scale insights into the mechanistic role of vacancy defects in governing bonding at the Ni/Al_2_O_3_ interface.

## 1. Introduction

Over the past few years, metal matrix ceramic composites (MMCCs) have gradually replaced traditional metallic materials in marine equipment applications, demonstrating broad prospects. Among them, Ni-Al_2_O_3_ composites have emerged as crucial functional coating materials due to their excellent corrosion resistance, high-temperature strength, and thermal stability [1,2,3]. Notably, the Ni/Al_2_O_3_ interface, as a critical region of the composite, not only performs essential functions in energy transfer and load distribution but also directly determines the overall performance of the composite material. However, harsh marine conditions (e.g., erosion and abrasion) impose increasingly demanding requirements on interfacial adhesion [4]. Therefore, investigating the microscopic mechanisms and optimization strategies of the Ni/Al_2_O_3_ interface holds significant scientific and practical importance.

To this day, Ni-Al_2_O_3_ composites highlight various fabrication methods to enhance their mechanical properties and corrosion resistance. For instance, Upadhyaya et al. [5] demonstrated that electroless Ni plating significantly improves the corrosion resistance of cold-sprayed Ni/Al_2_O_3_ interfaces by effectively mitigating chloride ion penetration. Building on this, Chang et al. [6] fabricated Ni-Al_2_O_3_ composite coatings via electrodeposition, revealing a direct proportional relationship between coating hardness and Al_2_O_3_ content. Further improving performance, Chen et al. [7] applied laser remelting to Ni-Al_2_O_3_ nanocomposite coatings, which simultaneously enhanced both tensile strength and elongation. In contrast, Dhand et al. [8] experimentally confirmed that thermally sprayed Ni-Al_2_O_3_ coatings exhibit superior wear resistance compared to their cold-sprayed counterparts. Beyond conventional reinforcements, Gong et al. [9] discovered that incorporating rare earth elements (Y, Ce) into ceramic/metal systems not only enhances corrosion resistance but also improves thermal shock resistance, offering a promising avenue for extreme-environment applications. However, these experimental approaches primarily provide empirical correlations without fully revealing the underlying atomic and electronic-scale mechanisms of the Ni/Al_2_O_3_ interface.

First-principles calculation methods, owing to their foundation in fundamental quantum mechanical principles, have become one of the most important theoretical approaches for investigating solid–solid interfaces [10,11,12]. Extensive studies have been conducted on metal–ceramic interfaces using this approach, revealing key insights into interface stability and bonding mechanisms [13,14,15,16,17,18]. For instance, Xue et al. [13] analyzed three interface configurations between Ti and different terminations (O, single-Al, and double-Al) of Al_2_O_3_ (0001), identifying the O-terminated interface as the most stable due to its ability to form stronger ionic bonds with Ti. Shi et al. [14] demonstrated that the O-terminated Al_2_O_3_ (0001)/Ni (111) interface exhibits the highest binding strength among the studied configurations. Liu et al. [15] further demonstrated that the O-terminated Ni/Cr_2_O_3_ interface represents the most stable configuration. Lin et al. [16] computationally showed that Al/TiC interfaces exhibit greater stability than Al/TiN interfaces. Beyond termination effects, researchers have explored broader material combinations. This electronic structure perspective was further quantified by Li et al. [17], who identified strong Fe-C ionic interactions as the primary contributor to enhanced adhesion strength in Fe/SiC interfaces through detailed charge density difference analysis. Bao et al. [18] found that dopant Yttrium substantially improves both the tensile strength and work of separation at Ni/Al_2_O_3_ interfaces. In fact, the interface region of a composite may contain various types of defects, such as vacancies, dislocations, and segregations. Based on this, the current work focuses specifically on investigating the influence of vacancy defects on properties of the Ni/Al_2_O_3_ interface.

Most recently, our team has systematically investigated the failure mechanisms and optimization strategies of ideal Ni/Al_2_O_3_ interfaces [19,20]. Based on these considerations, the present work selects the O-terminated Ni/Al_2_O_3_ interface, which exhibits the highest binding strength, as the primary research subject. The specific research content is as follows. Firstly, the γ-Ni (111)/α-Al_2_O_3_ (0001) interface structures with vacancies at different locations were constructed and optimized. Subsequently, the atomic relaxation behavior at the interface was analyzed to establish the relationship between vacancy positions and the bonding strength of the interface. Finally, the electronic structures of various interface configurations were examined to elucidate the influence of vacancy defects on interfacial bonding mechanisms. This study is expected to provide fundamental guidance for the interface theory of the Ni-Al_2_O_3_ composite.

## 2. Results and Discussion

### 2.1. Bonding Strength of the Interface

The work of adhesion (W_ad_) is defined as the energy required to separate a unit area of an interface between two dissimilar materials into two free surfaces [21]. This thermodynamic parameter provides a quantitative description of interfacial adhesion strength. Generally, a higher value of W_ad_ at the interface corresponds to greater bonding strength. To investigate the effect of vacancy defects on the Ni/Al_2_O_3_ interface, it is necessary to calculate the W_ad_ for both ideal and defective Ni/Al_2_O_3_ interface structures using Equation (1) [22]:

(1) $$W_{ad}=\frac{E_{Ni}+E_{{Al}_{2}O_{3}}-E_{Ni/{Al}_{2}O_{3}}}{A}$$

where E_Ni_ and E_Al2O3_ represent the energy of the Ni-only block and the Al_2_O_3_-only block, respectively, when the opposing surfaces of each model are replaced by vacuum layers. E_Ni/Al2O3_ is the total energy of the Ni/Al_2_O_3_ interface structure, and A denotes the interfacial area with the unit of m^2^.

**Figure 1:**
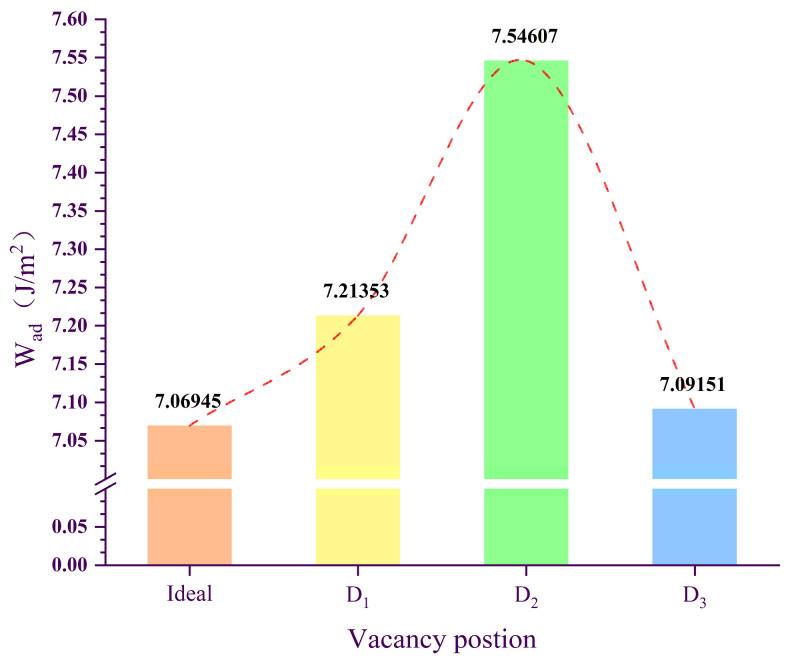
The work of adhesion of ideal and defective Ni/Al_2_O_3_ interface structures.

The results of W_ad_ for ideal and defect-containing Ni/Al_2_O_3_ interfaces are shown in Figure 1, with the following ranking: D_2_ (7.54607 J/m^2^) > D_1_ (7.21353 J/m^2^) > D_3_ (7.09151 J/m^2^) > Ideal (7.06946 J/m^2^). Herein, the structures corresponding to ideal, D1, D2, and D3 are provided in Figure 11 of Section 3.2. Compared to the ideal interface, the W_ad_ for the D_1_ and D_2_ structures shows a significant increase, rising by 2.0% and 6.7%, respectively, while the increase for the D_3_ structure is negligible. This demonstrates that first- and second-layer vacancy defects enhance interfacial bonding, whereas third-layer vacancies, located farther from the interface, have negligible effects on bonding strength. Related studies have confirmed that vacancy defects can positively influence interfacial properties. For instance, Chen et al. [23] revealed that graphene/Al interfaces with single-vacancy defects exhibit significantly higher W_ad_ than ideal interfaces. Similarly, Liu et al. [24] demonstrated that vacancy defects enhance the stability of Cu/graphene interfaces.

**Figure 2:**
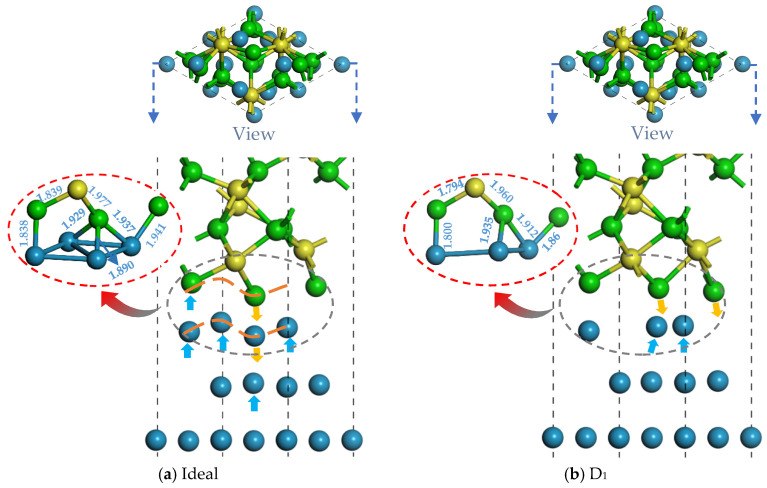

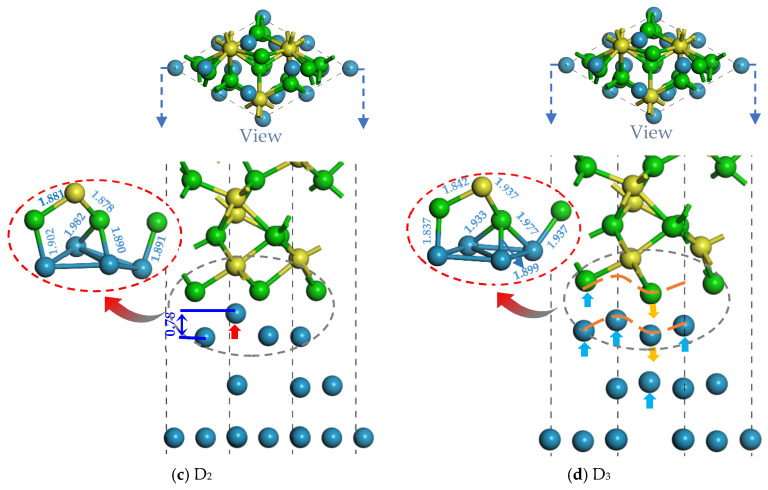
Local structure of ideal and defective Ni/Al_2_O_3_ interface after relaxation (unit of length/Å). Herein, (**a**) Ideal structure, (**b**) First-layer vacancy structure, (**c**) Second-layer vacancy structure, and (**d**) Third-layer vacancy structure.

Figure 2 displays the atomic structure of the ideal and defective Ni/Al_2_O_3_ interface after optimization. Relative to the ideal Ni/Al_2_O_3_ interface, the vacancies can induce varying degrees of atomic migration at the interface, which microscopically reflects the balanced interatomic interactions. In particular, it can be observed that the bond length and bond angle between atoms in the interface region change, which indicates that the formation of vacancies changes the microstructure of the interface. The change in these structural parameters confirms that vacancy defects fundamentally reconstruct the atomic configuration of the interface by inducing local lattice distortion. As shown in Figure 2b, the D_1_ vacancy configuration exhibits modified interfacial relaxation due to missing first-layer Ni atoms. Both O and Ni atoms at the interface migrate toward the interfacial center, indicating enhanced interatomic interactions within the defect-containing region. For the D_2_ structure (Figure 2c), it demonstrates that the vacancy causes the release of constraints on the first layer of Ni atoms, which move toward the center of the interface by 0.78 Å. This phenomenon reduces the interfacial spacing, thereby enhancing the bonding strength between Ni and Al_2_O_3_. Notably, a collective rightward displacement occurs in the top three atomic layers of the Ni substrate. In Figure 2d, the D_3_ structure shows an overall parallel movement of the interface layer atoms, while the interface spacing does not change (see red dotted line). In fact, this microstructure reconstruction will directly affect the electron density distribution of the interface system and then change the composition and distribution characteristics of the interface binding energy.

This phenomenon demonstrates that vacancies in the first and second atomic layers of the Ni significantly influence interfacial bonding, while their effects diminish with increasing depth. In summary, near-surface vacancy defects in the Ni matrix positively influence the interface properties, which aligns with the results of W_ad_ above. These findings collectively explain the variations in the interfacial bonding strength across different vacancy configurations, establishing a clear structure–property relationship between vacancy geometry and interface performance. To reveal the underlying mechanisms, further electronic-scale analysis will be conducted on the interfaces with vacancy defects, focusing on charge density redistribution and bonding orbital interactions.

### 2.2. Electronic Properties of Interface

Generally, the distribution and transfer of electrons at the interface can regulate the breaking and reconstruction of chemical bonds, thereby governing the interfacial bonding mechanism. Accordingly, this research discusses the electronic dynamics at the ideal and defective Ni/Al_2_O_3_ interface, including charge density, charge density difference, overlap population of the bond, and density of states.

**Figure 3:**
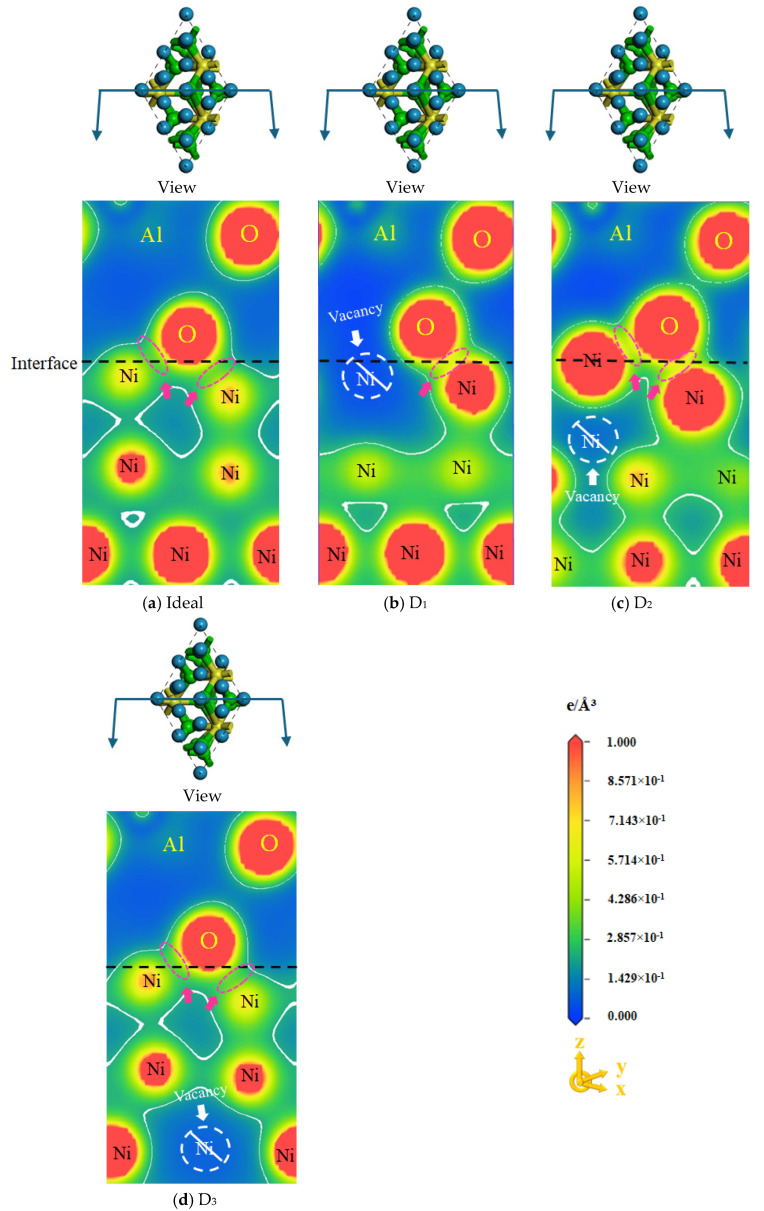
Charge density of ideal and defective Ni/Al_2_O_3_ interface structures. Herein, (**a**) Ideal structure, (**b**) First-layer vacancy structure, (**c**) Second-layer vacancy structure, and (**d**) Third-layer vacancy structure.

Figure 3 displays the charge density of ideal and defective Ni/Al_2_O_3_ interfaces, where the color gradient from blue to red indicates progressively increasing charge concentrations. At non-interface regions, the charge distribution concentration in the atomic gap of the Ni matrix is significantly stronger than the Al_2_O_3_ side, which is determined by their respective crystal structure characteristics. The non-uniform charge-sharing zones between the Ni and O atoms are observed at the interface, indicating that vacancy defects induce different degrees of dynamic movement of electrons. Compared to the ideal Ni/Al_2_O_3_ interface, the D_1_ and D_2_ structures (Figure 3b,c) exhibit a marked increase in electron density between Ni and O atoms at the interface, as evidenced by the color gradient. Particularly, the D_2_ interface displays the strongest charge density, whereas D_3_ approaches ideal interface characteristics. Indeed, the charge concentration of the interface can represent the strength of the covalent bond. Therefore, the higher the charge density at the interface, the stronger the bonding strength of the interface, which is also consistent with the above W_ad_.

**Figure 4:**
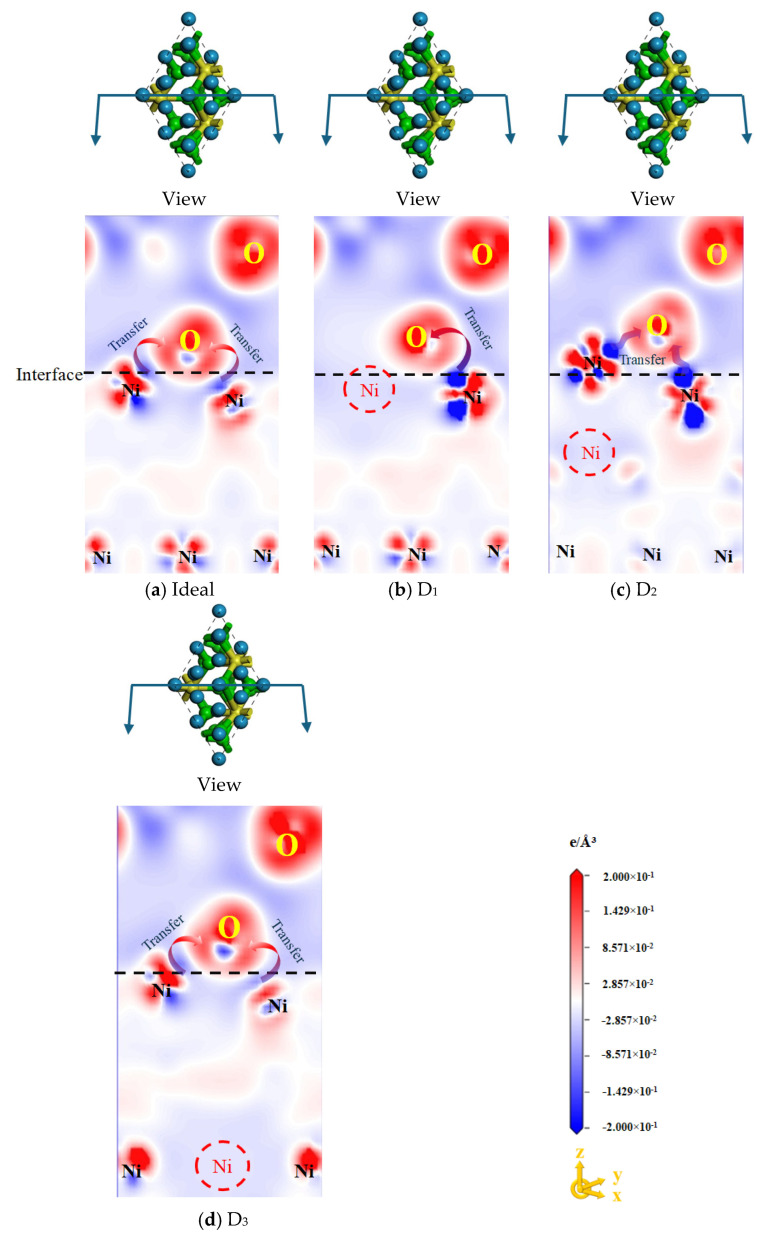
Charge density differences in ideal and defective Ni/Al_2_O_3_ interface structures. Herein, (**a**) Ideal structure, (**b**) First-layer vacancy structure, (**c**) Second-layer vacancy structure, and (**d**) Third-layer vacancy structure.

To further analyze electron transfer behavior at the Ni/Al_2_O_3_ interface, differential charge density was calculated for both ideal and defective structures, as shown in Figure 4. Here, the red-colored areas correspond to electron accumulation, whereas the blue-colored areas represent electron dissipation. The comparative analysis in Figure 4a–c demonstrates that the Ni atoms at the D_1_ and D_2_ interfaces form a deeper electron depletion region while the O atoms exhibit a more pronounced charge aggregation region relative to the ideal Ni/Al_2_O_3_ interface. This indicates that electrons are transferred from Ni to O atoms, forming a Ni-O ionic bond. Taking into account the observed charge density distribution, it can be inferred that the Ni/Al_2_O_3_ interface is predominantly characterized by ionic Ni–O bonds, with a minor contribution from covalent interactions. The presence of vacancies alters the local atomic structure, leading to changes in interlayer spacing and subsequent modifications in chemical bond strength, thereby affecting interfacial properties. Previous studies [14] have also confirmed a direct correlation between the charge distribution of Ni–O bonds and interlayer spacing at the Ni/Al_2_O_3_ interface.

In other words, the bond strength of Ni-O at the D_1_ and D_2_ interfaces is significantly greater than that at the ideal interface. In particular, the Ni-O ionic bond characteristic of the D_2_ interface is the most prominent. As evidenced in Figure 4d, the D_3_ interface exhibits an electron transfer situation similar to that of the ideal interface, indicating that vacancy defects away from the surface have a negligible effect on the electron transfer at the interface. In summary, the charge transfer characteristics observed across vacancy-modified interfaces exhibit strong consistency with the corresponding result of W_ad_, collectively validating the causal relationship between defect-induced electronic redistribution and interfacial bonding enhancement.

**Figure 5:**
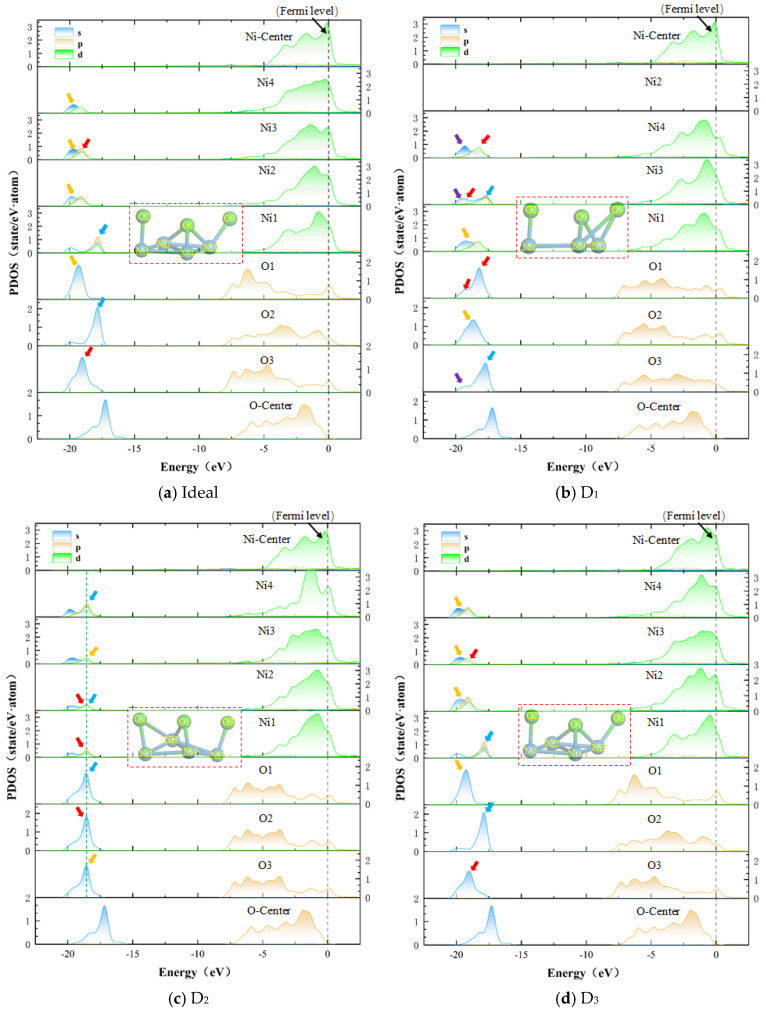
The PDOS of ideal and defective Ni/Al_2_O_3_ interface structures (arrows of the same color represent the bonding peaks between different atoms). Herein, (**a**) Ideal structure, (**b**) First-layer vacancy structure, (**c**) Second-layer vacancy structure, and (**d**) Third-layer vacancy structure.

To gain deeper insights into the influence of vacancy defects on the bonding mechanism at the Ni/Al_2_O_3_ interface, it is essential to investigate the hybridization behavior of electronic orbitals through projected density of states (PDOS). Figure 5 presents the PDOS plots for ideal and defective Ni/Al_2_O_3_ interfaces, and the black dashed line indicates the Fermi level. In all interfacial configurations, the PDOS of interfacial O atoms exhibits a pronounced peak above zero at the Fermi level (marked by black dashed lines), in contrast to bulk O-center atoms, demonstrating that the O atoms at the interface have been metallized by Ni, forming partial metallic bonds. The PDOS spectrum of the D_1_ structure is shown in Figure 5b. In the energy range of −20 to −17.5 eV, the PDOS peaks of Ni and O atoms at the interface exhibit significant shape similarity (as indicated by the colored arrows). This corresponds to a typical bonding peak, indicating that electron orbitals are hybridized between atoms. It is worth noting that compared to the ideal Ni/Al_2_O_3_ interface, the D_1_ structure resulted in a greater number of bonding peaks, which means enhanced electron orbital hybridization at the interface, resulting in stronger covalent bonds. Previous studies have demonstrated that a larger overlap area of hybrid orbital bonding peaks at the interface correlates with higher bonding strength of the interface [25]. Further analysis of Figure 5c reveals that the D_2_ structure exhibits a greater overlap area of the bonding peak compared to the ideal interface, suggesting a corresponding enhancement in the bonding strength of the interface. In contrast, the PDOS spectrum of the D_3_ structure (Figure 5d) shows no significant differences. In fact, the electron orbital hybridization between atoms at the interface serves as the microscopic manifestation of interfacial reactions, which aligns with Chun et al.’s [26] conclusion that the Ni/Al_2_O_3_ interface produces NiO and Ni_x_Al_1-x_. However, the Ni/Al_2_O_3_ interface with vacancy defects can modulate the strength of electron orbital hybridization, leading to variations in the types and quantities of interfacial reaction products, thereby affecting the bonding properties of the interface. These results demonstrate the first- and second-layer vacancy-driven bonding strength of the interface by increasing the degree of electron orbital hybridization at the interface, thereby exhibiting superior bonding characteristics.

**Figure 6:**
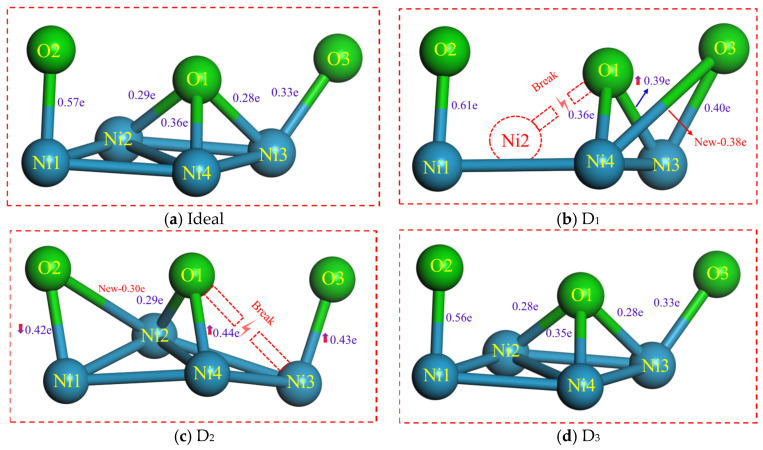
The overlap population of bonds for the ideal and defective Ni/Al_2_O_3_ interface. Herein, (**a**) Ideal structure, (**b**) First-layer vacancy structure, (**c**) Second-layer vacancy structure, and (**d**) Third-layer vacancy structure.

In order to directly quantify the binding strength of the Ni/Al_2_O_3_ interface, the electron overlap population (EOP) of the bonds between the Ni and O atoms at the interface is calculated, as shown in Figure 6. Positive values of EOP represent a covalent character, with larger values indicating stronger chemical bonds [27]. As shown in Figure 6b, the D_1_ structure shows a 39.3% increase in the EOP value of O1-Ni3 bonds compared to that of the ideal interface. For the D_2_ structure (Figure 6c), the EOP value of O2-Ni1 decreased by 26.3%, whereas the O1-Ni4 and O3-Ni3 pairs exhibited increases by 22.2% and 30.3%, respectively. It can therefore be inferred that the formation of vacancies at the interface induces electron transfer between atoms. An increase in the EOP value signifies enhanced interatomic interaction, corresponding to a strengthening of interfacial bonding. Another study [28] demonstrated that a decrease in the EOP value of the metal–ceramic interface region leads to the failure of the structure. Interestingly, both D_1_ and D_2_ structures exhibit not only increased total EOP but also the breaking of old bonds and the formation of new bonds, likely caused by lattice distortion induced by vacancy defects. In contrast, the D_3_ structure shows negligible variation. Thus, electron distribution and transfer constitute the fundamental determinants of the bonding performance of the Ni/Al_2_O_3_ interface.

In summary, the effects of vacancy defects on Ni/Al_2_O_3_ interfaces operate through three synergistic mechanisms. First, the atomic relaxation induced by vacancy defects significantly alters the local geometric configuration. Second, charge redistribution leads to a remarkable enhancement of interfacial electron density. Third, the strengthened orbital hybridization effect at the interface further intensifies covalent interactions. These collective behaviors jointly induce variations in the W_ad_ at the Ni/Al_2_O_3_ interface, thereby enhancing its interfacial bonding strength. This discovery not only deepens our understanding of interface behavior in metal–ceramic composites but also provides new theoretical foundations for optimizing a composite interface design. Based on these findings, we plan to systematically investigate the effects of different defect types (e.g., interstitial atoms and dislocations) on interfacial properties, aiming to establish comprehensive theoretical guidance for developing high-performance composite materials.

## 3. Calculation Method and Details

### 3.1. Calculation Settings

All first-principles calculations in this work, encompassing bulk, surface, and interface behaviors, were conducted using the density functional theory (DFT)-based Cambridge Sequential Total Energy Package (CASTEP) [29,30]. Ultrasoft pseudopotentials (USPPs) [31] were employed to enhance computational efficiency in treating electron-ion interactions. To obtain the ground state configuration, the Broyden–Fletcher–Goldfarb–Shannon (BFGS) [32,33] algorithm was used for atom structures. The calculation parameters are set as follows. The Perdew–Burke–Ernzerhof (PBE) functional within the generalized gradient approximation (GGA) [34] framework was employed as the exchange–correlation functional to describe the Ni/Al_2_O_3_ interface system. 

**Figure 7:**
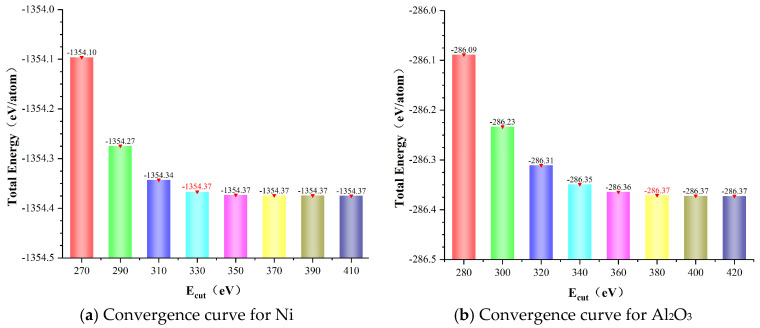
Convergence verification of total energy for different E_cut_. (**a**) Ni and (**b**) Al_2_O_3_.

**Figure 8:**
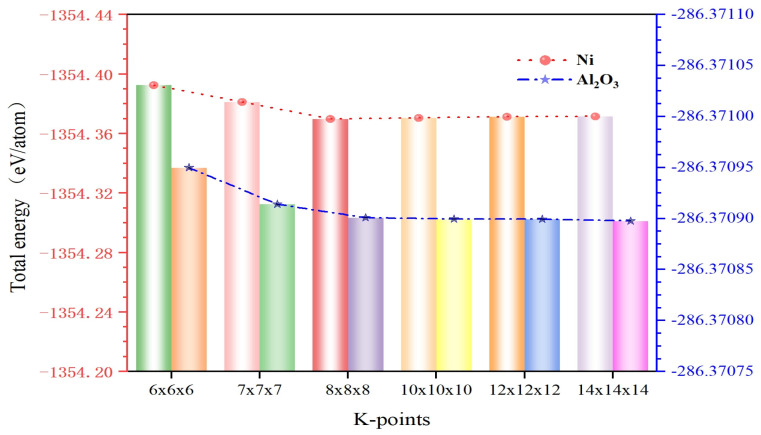
Convergence verification of total energy for different k-points.

Here, plane-wave cutoff energy and k-point sampling density were carried out by convergence tests, as shown in Figure 7 and Figure 8. Thus, the cutoff energy was set to 380 eV with a k-point mesh of 8 × 8 × 1 for Ni/Al_2_O_3_ interface systems.

**Figure 9:**
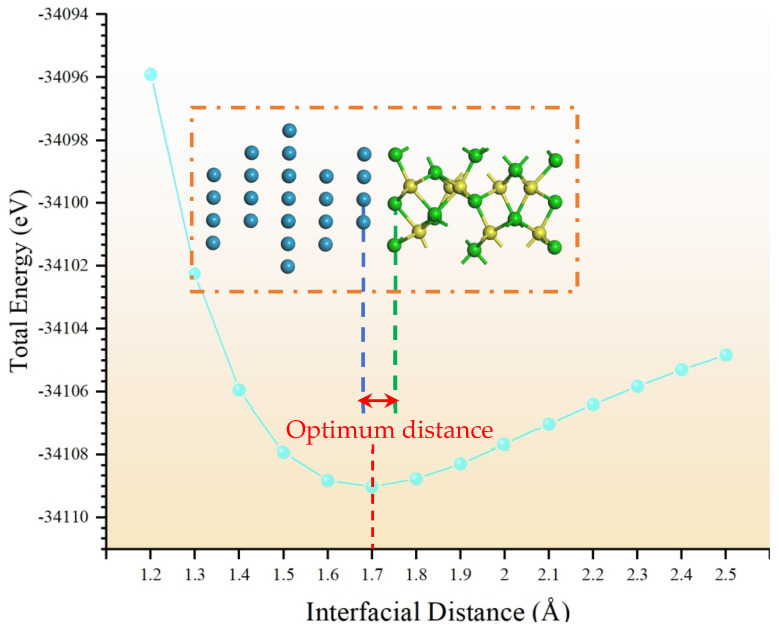
The relationship between total energy and interfacial distance for the Ni/Al_2_O_3_ structure.

Additionally, the total energy was calculated as a function of interface distance. As shown in Figure 9, the structure is most stable when the interface distance is 1.7 Å. Furthermore, the convergence criteria were set as follows: energy change tolerance of 1.0 × 10^−5^ eV/atom, maximum force below 0.03 eV/Å, and maximum displacement of less than 0.001 Å. These parameters ensured rigorous geometric convergence of all systems.

### 3.2. Model Building

**Figure 10:**
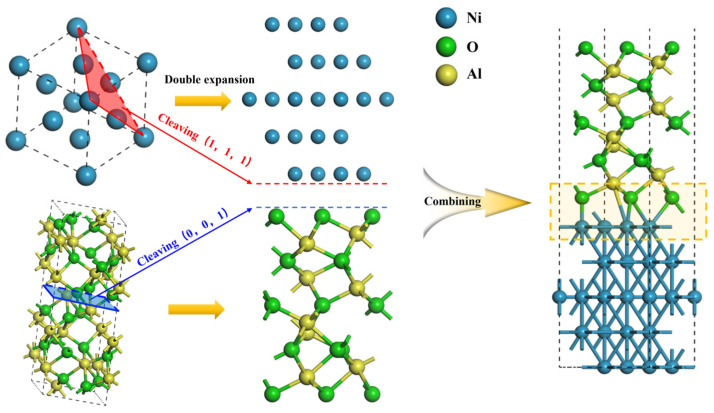
The establishment process of Ni(111)/Al_2_O_3_(0001) interface configuration.

Previous experimental studies [35] have confirmed that stable interfaces in heterogeneous materials tend to form between surfaces with the lowest surface energy. Accordingly, the Ni (111) [36] and Al_2_O_3_ (0001) [37] surfaces were selected for this study. It should be noted that the Al_2_O_3_ (0001) surface exhibits three distinct terminations: 1Al-, 2Al-, and O-terminated configurations. Our earlier work [19] demonstrated that the characteristic properties of Al_2_O_3_ can be accurately captured with atomic layer thicknesses of 12, 14, and 13 layers for the 1Al-, 2Al-, and O-terminated Al_2_O_3_ (0001) surfaces, respectively. Similarly, Ni (111) requires at least five atomic layers to adequately represent its bulk characteristics [36]. However, the most stable interface configuration is achieved between a 5-layer Ni (111) surface and a 13-layer O-terminated Al_2_O_3_ (0001) surface. Based on these considerations, we constructed three distinct Ni-Al_2_O_3_ interface models as illustrated in Figure 10.

**Figure 11:**
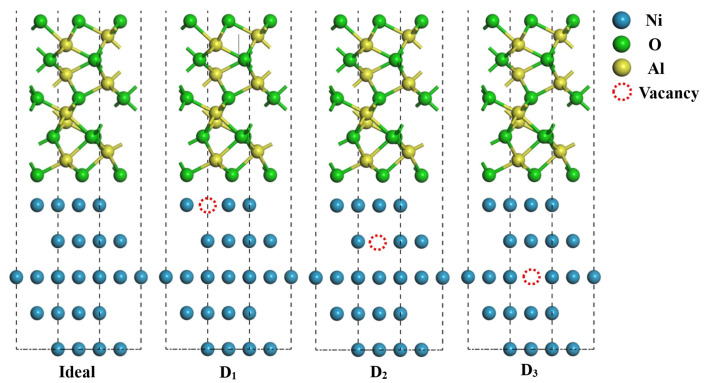
The ideal and defective Ni/Al_2_O_3_ interface structures.

According to the methodology established by Zhang et al. [38], a systematic comparative analysis was conducted by maintaining a constant vacancy concentration of 25% while strategically varying the spatial distribution of vacancy sites. This involves the introduction of vacancy defects in the first, second, and third atomic layers of Ni (hereafter referred to as D_1_, D_2,_ and D_3_, respectively), as shown in Figure 11.

## 4. Conclusions

This study systematically investigates the atomic and electronic properties of Ni (111)/Al_2_O_3_ (0001) interface structures modified by vacancy defects at different sites, focusing on the role of vacancies in interfacial bonding mechanisms.

(1) The results of W_ad_ indicate that vacancy defects have a positive effect on the bonding performance of Ni/Al_2_O_3_ interfaces. Specifically, vacancy defects in the first (D_1_) and second (D_2_) layers of Ni increase the W_ad_ by 2% and 6.7%, respectively, whereas the influence diminishes with increasing vacancy-defect depth.

(2) Various types of modified vacancy defects can induce atomic relaxation behaviors at the Ni/Al_2_O_3_ interface. For the D_1_ and D_2_ structures, the atoms at the surface move toward the center of the interface and shorten the interface spacing, which in turn increases the binding strength of the interface. Note that the atoms at the D_3_ interface move in parallel and do not change the interface spacing. Therefore, the occurrence of atomic relaxation behavior is a microscopic manifestation of the release of stress, making the Ni/Al_2_O_3_ interface structure more stable.

(3) The formation of vacancies alters the charge concentration in the shared region of the Ni/Al_2_O_3_ interface. The D_1_ and D_2_ structures exhibit significantly increased charge concentration, indicating electron transfer from both sides to the interface region. Specifically, the D_2_ structure exhibited the highest charge density, resulting in the strongest bonding strength of the interface.

(4) Vacancy defects can influence the hybridization behavior of electron orbitals at the interface. The D_1_ and D_2_ structures enhance the bonding strength of the interface by promoting electron orbital hybridization between Ni and O atoms at the Ni/Al_2_O_3_ interface, specifically manifested as an increase in the number of bonding peaks and an enlargement of the overlapping area of bonding peaks in the PDOS spectra. The greater the electron orbital hybridization, the stronger the bonding strength of the interface.

## Data Availability

The original contributions presented in this study are included in the article. Further inquiries can be directed to the corresponding author.
